# Risk factors of degenerative lumbar scoliosis in patients with lumbar spinal canal stenosis

**DOI:** 10.1097/MD.0000000000017177

**Published:** 2019-09-20

**Authors:** Chunlei Wang, Hengrui Chang, Xianda Gao, Jiaxin Xu, Xianzhong Meng

**Affiliations:** a Department of Spine Surgery, The Third Hospital of HeBei Medical University; b Key Laboratory of Biomechanics of Hebei Province, Shijiazhuang, Hebei, P.R. China.

**Keywords:** degenerative lumbar scoliosis, degenerative lumbar spinal stenosis, risk factors

## Abstract

Lumbar spinal canal stenosis (LSCS) associated with degenerative scoliosis has being increasingly aware by the public and studied by many researchers. Degenerative changes leading to spinal stenosis can precede a spinal deformity which will develop into the de novo scoliosis. There are few studies focusing on the risk factors contributing to the degenerative lumbar scoliosis (DLS) in lumbar spinal stenosis patients.

From September, 2017 to December, 2017, 181 patients who were diagnosed with LSCS in the outpatient department of our hospital were enrolled in this retrospective investigation. The patients were divided into 2 groups: DLS group (Cobb angle >10°) and LSCS group. Sex, age, smoking status (yes or no), occupation (heavy or light labor), body mass index (BMI), bone mineral density (BMD) and radiographic parameters including the lumbar lordosis (LL), pelvic incidence (PI), pelvic tilt (PT), sacral slope (SS), thoracic kyphosis (TK), coronal vertical axis, and sagittal vertical axis (SVA) are all evaluated as potential risk factors. Multivariate logistic regression analysis and receiver-operating characteristic curves were used to identify potential risk factors.

Forty-five of 181 patients were diagnosed with DLS and involved in the DLS group. There were significant differences between the 2 groups in BMI (*P* < .001), LL (*P* = .0046), BMD (*P* < .001), SVA (*P* < .001), and TK (*P* = .047). BMD < −1.85 g/cm^2^ (adjusted odds ratio [AOR] 0.030, 95% confidence interval [CI] 0.008–0.107, *P* < .001), BMI >25.57 kg/m^2^ (AOR 1.270, 95% CI 1.040–1.551, *P* = .019), and SVA >3.98 cm (AOR 3.651, 95% CI 2.226–5.990, *P* < .001) had good accuracy to predict the formation of degenerative lumbar scoliosis based on degenerative lumbar spinal stenosis.

Degenerative lumbar scoliosis has a high incidence in degenerative lumbar spinal stenosis. BMD <−1.85 g/cm^2^, BMI >25.57 kg/m^2^, and SVA >3.98 cm were the potential risk factors for the formation of degenerative lumbar scoliosis based on degenerative lumbar spinal stenosis.

## 1 Introduction

Lumbar spinal canal stenosis (LSCS) is a common problem in the elderly people, and is a common clinical symptom characterized by back pain and/or lower-extremity neurological symptoms. However, patients with lumbar stenosis are always adopting a forward bending posture to relieve neural compression,^[1]^ which is an attempt to increase the volume of the central vertebral canal and the intervertebral foramina. Clinically, patients with a forward bending posture may have the similar symptom with the patients having sagittal spinal deformity. However, we found that some patients existed degenerative lumbar scoliosis (Cobb angle >10°), and some patients simply had lumbar stenosis with the discovery of the disease. Degenerative lumbar scoliosis is due to its bony mature after some degenerative element prompted a side of the spine with the scoliosis deformity,^[2,3]^ which can cause low back pain, radiating pain in lower limbs, and neurogenic claudication, thus having a considerable impact on their daily lives. But most people only have limited knowledge about the risk factors associated with lumbar scoliosis, especially in the elderly patients with spinal stenosis. So the purpose of this article is to deeply analyze the potential risk factors associated with the development and progression of degenerative lumbar scoliosis (DLS) in patients with LSCS.

## 2 Material and methods

### 2.1 Patients

From September, 2017 to December, 2017, 181 participants who were diagnosed with LSCS visited the outpatient department of our hospital. We have used cross-sectional analysis to determine the risk factors for DLS in patients with LSCS, which was also approved by Institution Review Board of HeBei Medical University.

The inclusion criteria for this study as follows: aged older than 50 years; the presence of back pain and/or lower-extremity neurological symptoms (radiating pain in lower limbs and/or neurogenic claudication, etc); and complete imaging data. The subjects were removed if they have the following characteristics: spine fractures, spinal surgery, or spinal tumor; spondylolisthesis, cervical spinal stenosis, and/or thoracic spinal stenosis; neurological deficit, muscular atrophy, neuromuscular diseases; lower-extremity vascular disease; and inflammation or infections. Patients were listed into the DLS group if they were diagnosed with LSCS having Cobb angle more than 10°. Patients were listed into the LSCS group if their primary presenting complaint was caused by spinal canal stenosis without DLS comprised patients with <10° Cobb angle. Sex, age, smoking status (yes or no), occupation (heavy or light labor), body mass index (BMI), bone mineral density (BMD), and radiographic parameters including the lumbar lordosis (LL), pelvic incidence (PI), pelvic tilt (PT), sacral slope (SS), thoracic kyphosis (TK), coronal vertical axis (CVA), and sagittal vertical axis (SVA) are all evaluated as potential risk factors for the formation of degenerative lumbar scoliosis based on degenerative lumbar spinal stenosis.

### 2.2 Radiological evaluation

**Figure 1:**
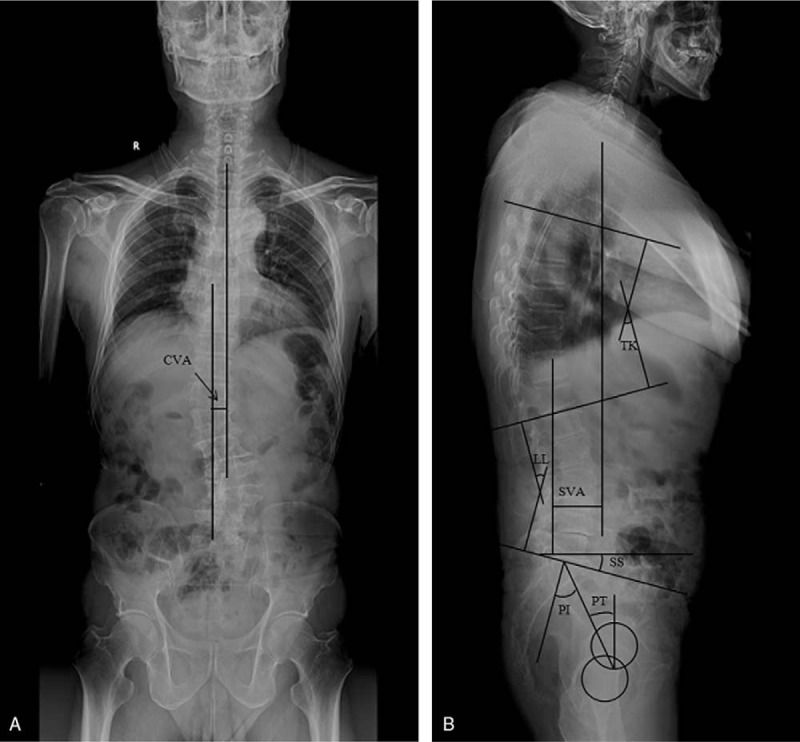
Illustration shows the radiographic measurements of spinopelvic parameters included in this analysis. A 57-year-old female patient who has clinical characterized by back pain, radicular or claudicant leg pain, and disability. she has a long history of smoking for 7 years with 20 cigarettes a day.

All the patients have taken the anteroposterior and lateral radiographs of the whole spine. Magnetic resonance imaging of the lumbar spine was performed with a 1.5-T imager (Vision; Siemens Medical Solutions, Erlangen, Germany). The entire lumbar spine was studied on the sagittal images (T1–S1) including parasagittal imaging of all the neural foraminae bilaterally (Fig. 1). Radiological parameters were recorded (Fig. 1), including LL, the angle between the lower end plate of L1 and the lower end plate of L5 on frontal radiographs; PT, the angle between a line drawn from the S1 endplate to the center of the femoral heads drawn intersecting the femoralheads; PI, the angle subtended by a line connecting the center of the femoral head to the center of the cephalad end plate of S1 and a second line drawn perpendicular to the S1 endplate at its center; SS, the angle between a line drawn parallel to the S1 endplate and the horizontal plane; and TK, the angle between the upper end plate of the T5 and the lower end plate of the T12. SVA is defined as the horizontal distance between the C7 plumb and the posterosuperior corner of the sacrum, which if >5 cm was used to regarded as sagittal imbalance^[4,5]^ (Fig. 1). CVA is defined as the horizontal distance between the C7 plumb and central sacral vertical line, which if >5 cm was used to regarded as coronal imbalance^[4,5]^ (Fig. 1). If the femoral heads do not overlap, the midpoint of the line connecting the centers of the femoral heads is considered to be the center of the femoral head (Fig. 1). Each datum was measured twice by the same author for 2 weeks, and the average was recorded and calculated for analysis.

### 2.3 Statistical analysis

All statistical analyses of data from both groups were conducted by the SPSS system (version 22.0). The Shapiro-Wilk test was used to determine whether data were normally distributed. Categorical variables were measured as numbers or as percentages of patients. Continuous variables were compared using the Mann–Whitney *U* test or an independent Student *t* test. Categorical data were assessed using a chi-square test. Logistic regression analysis with the occurrence of DLS as an objective factor was also performed to identify the risk factors for the occurrence of DLS. Binary logistic regression was performed to assess the impact of all potential predictors (significant in the univariate analysis) on DLS using forward model. Adjusted odds ratio (AOR), 95% confidence interval (CI), and *P* values were provided for each predictor. Eventually, logistic regression analysis was again used to determine the relationship between lumbar scoliosis and predictors based on degenerative lumbar spinal stenosis. A *P* value <.05 was considered as statistically significant.

## 3 Results

Forty-five of 181 patients were diagnosed with DLS and involved in the DLS group (mean age 59.40 ± 6.00 years, 15 males and 30 females) and 136 patients in LSCS group (mean age 59.87 ± 6.19 years, 43 males and 93 females) (Table 1).

**Table 1:**
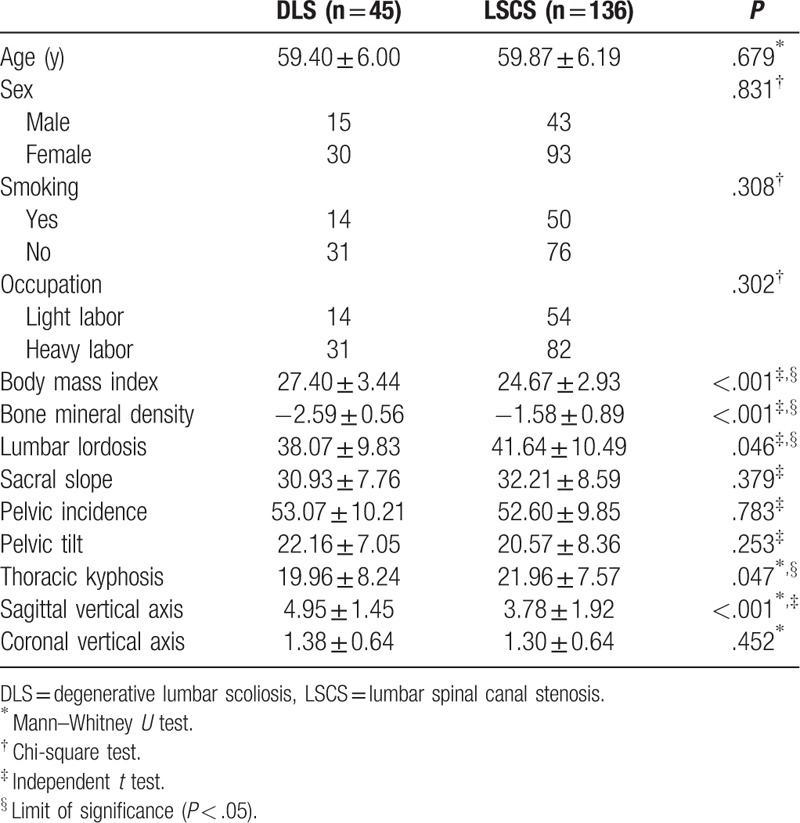
Comparison of preoperative patient characteristics between DLS group and LSCS group.

Comparison of patient characteristics between the 2 groups is shown in Table 1. There were statistically significant differences between the 2 groups regarding BMI (*P* < .001), LL (*P* = .046), BMD (*P* < .001), SVA (*P* < .001), and TK (*P* = .047). In our study, we found there are more female patients included in the DLS group than the LSCS group (*P* = .831). There is a nonsignificant trend toward a higher PT (22.16 ± 7.05 vs 20.57 ± 8.36°; *P* = .253) and PI (53.07 ± 10.21 vs 52.60 ± 9.85°; *P* = .783) in the DLS group. Univariate regression analysis showed statistically significant differences in BMI (*P* < .001), BMD (*P* < .001), LL (*P* = .048), TK (*P* = .027), and SVA (*P* < .001). Furthermore, 3 factors in multivariate logistic regression analysis were selected as potential risk factors: BMD (AOR 0.032, 95% CI 0.010–0.104, *P* < .001), BMI (AOR 1.254, 95% CI 1.037–1.517, *P* = .020), and SVA (AOR 3.643, 95% CI 2.241–5.922, *P* < .001) (Table 2).

**Table 2:**
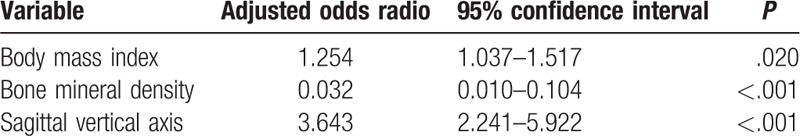
Multiple logistic regression analysis with occurrence of degenerative lumbar scoliosis as objective factor.

The receiver-operating characteristic (ROC) curve analysis shows that BMI (area under the curve 0.735, *P* < .001), BMD (area under the curve 0.825, *P* < .001), and SVA (area under the curve 0.683, *P* < .001) were potential risk factors have good accuracy (Table 3).

**Table 3:**
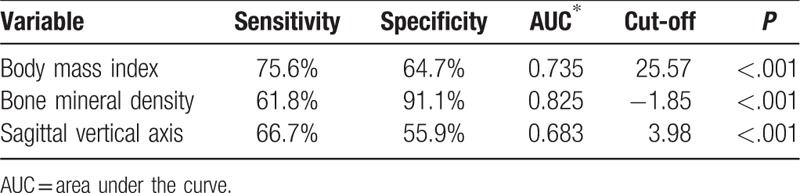
Sensitivity, specificity, AUC, and cut-off of predictors.

## 4 Discussion

Degenerative lumbar scoliosis now is becoming a common problem and should be paid more attention due to the aging population, increased capacity, and people's willingness to manage difficult problems for elderly patients. It is a very complex pathology as it often involves the intersection of degenerative lumbar spinal stenosis and spinal deformity.^[2,6]^ Although the etiology is not clear, it is truly associated with progressive and asymmetric degeneration of the disc, sex, facet joints, vertebral rotation, changes in LL and disc height, lumbar sagittal imbalance, and other structural spinal elements, which can typically lead to neural element compression.^[7–11]^ The findings of this study suggest that BMD <−1.85 g/cm^2^, BMI >25.57 kg/m^2^, and SVA >3.98 cm were the potential risk factors for the formation of degenerative lumbar scoliosis based on degenerative lumbar spinal stenosis.

In the present study, there was a statistically significant difference in BMD between the 2 groups (*P* < .001). It was found that BMD <−1.85 g/cm^2^ played a significant role in the formation or promotion of degenerative lumbar scoliosis progression in this study. Osteoporosis and degenerative scoliosis are frequently occurring diseases in older people, and degenerative scoliosis patients are accompanied by reduced bone density or osteoporosis.^[2,12]^ Osteoporosis could lead to low bone strength reduced adaptability to work load and increase the risk of osteoporotic vertebral compression fractures. Osteoporosis combined with vertebral fractures will result in anterior, middle, and posterior vertebral heights change, so subsequent changes in vertebral heights can lead to changes in the spinal curvature and to a redistribution of forces upon the vertebrae endplates, and degenerative curves become progressive as a result of the asymmetric load on weakened vertebrae, which becomes progressively more wedged and deformed.^[13]^ High asymmetry loss due to vertebral compression changes the normal alignment of the spine, leading to kyphosis, sagittal plane deformity, and potential coronal imbalance.^[14]^ Osteoporosis has been implicated in the development of degenerative scoliosis in the adult, as it has been shown that patients with osteoporosis are more likely to exhibit scoliosis.^[3]^ However, some reports indicate that scoliosis predisposes to osteoporosis,^[15]^ whereas other authors believe that osteoporosis predisposes to scoliosis,^[16]^ or that there is no correlation.^[17]^ Previous studies have shown that lumbar adult scoliosis is associated with low femoral neck BMD, but not with low vertebral BMD,^[16]^ and osteoporosis as such does not influence the curve magnitude or complication rates.^[18]^

Degenerative lumbar scoliosis is more common in postmenopausal women than men, possibly due to low levels of estrogen and progestin, increasing the risk of osteoporosis in many postmenopausal women.^[5,19]^ Previous studies have shown that DLS is a disorder of the adult spine with a distinct female predominance; it is approximately twice as common in women.^[17]^ In our study, there is an obvious trend that female patients may have the high occurrence of DLS. Quante et al^[20]^ also reported that osteoporosis can cause degenerative lumbar asymmetry, so that the obesity become asymmetric and gradual scoliosis.

Body mass index is an objective and simple indicator, as the values >25 and 30 kg/m^2^ can define overweight and obesity, respectively. Liuke et al^[21]^ have the evidence that BMI >25 kg/m^2^ can raise the risk of lumbar disk degeneration. Our study showed that BMI is closely related to the formation or progression of degenerative lumbar scoliosis, especially when the BMI is greater than 25.57 kg/m^2^, and it can be explained by 2 possible reasons. On the one hand, increased loading of the spine causes the intervertebral discs loss its height and reduce the ability to absorb the force of gravity, thus leading to an exceptional load around the facet joints and spinal ligaments. If obesity becomes a burden for a long time on the spine, it will lead to systemic inflammatory changes by the release of adipocytokines. It may affect the musculoskeletal system initiating degenerative changes in the vertebral column.^[22]^ On the other hand, the obesity patients with paraspinal muscle strength is weaker compared with the healthy adults. If the muscles do not have enough power to keep upright posture, it may accelerate the degeneration of disc and articular process. With these changes in intradiskal pressure, obesity can potentially give more pressure, accelerating the degenerative process of disc, which will lead to the formation of DLS. Obesity may not only be a risk factor related to the natural degradation of spine, but may also play a promoting role in occurrence of DLS. Therefore, reasonable control body weight pre-to-postoperative could cut down the rate of DLS.

Degenerative lumbar scoliosis not only exists with coronal imbalance, but also has changes in the sagittal plane rotational deformity of the spine. If the speed of the 2 sides were different, the unbalanced degeneration will result in scoliosis.

In this study, we used ROC analysis to find that SVA 3.98 cm can be used as another optimal cut-off point and SVA >3.98 cm is used to predict the formation or progression of degenerative lumbar scoliosis. SVA is an important indicator for assessing the sagittal balance of preoperative or postoperative surgery in patients with adult scoliosis.^[4,5,23]^ Djurasovic and Glassman^[24]^ studied the spine curve pattern, curve amplitude, coronal balance, sagittal balance, and apical rotation of 298 adults with scoliosis, and concluded that sagittal balance is the most reliable predictor of symptoms.

Previous researches have already reported lower SS^[25]^ and LL,^[8]^ and in degenerative lumbar scoliosis patients, which may be involved in the pathogenesis of DLS,^[26]^ which may, in turn, be closely related to clinical manifestations.^[27]^ Weishi et al^[28]^ reported that patients with the increment of PI and LL will gradually decrease, and the apex of the lordosis will also be raised.

In our study, we draw a similar conclusion that the mean values of LL in DLS group was lower than that in LSCS group (*P* = .046), and patients in DLS group had lower SS than in LSCS group; the difference was not statistically significant (*P* = .379), but there was a nonsignificant trend toward a higher PT (*P* = .253) and PI (*P* = .783) in the DLS group. It is recommended that the pelvis of patients with DLS adjust their posture by tilting backwards and backwards to compensate for sagittal imbalance. The correlation between LL and other sagittal parameters indicates that lumbar scoliosis and severe degeneration remain the ability to regulate the sagittal balance.

The present study has some limitations. First, only the Chinese Han individuals were involved in this study. Second, it is that only a small number of subjects were investigated, and the study may not be enough to determine some of the significant risk factors. Third, previous studies have shown that PI, as a pelvic morphological parameter, remains stable in adulthood after bone maturity,^[29]^ but recent studies have reported changes in PI.^[30]^ Therefore, some work should be done to find out if the PI differences in this study are from diseases or other age-related factors. Hence, these limitations indicate the need for large sample and multicenter studies to verify results.

## 5 Conclusions

Degenerative lumbar scoliosis has a high incidence in degenerative lumbar spinal stenosis. BMI >25.57 kg/m^2^, BMD <−1.85 g/cm^2^, and SVA >3.98 cm were the potential risk factors for the formation of degenerative lumbar scoliosis based on degenerative LSCS. More attention should be paid how to slow down osteoporosis with age and to properly control weight to avoid degenerative scoliosis, especially if the 3 potential risk factors are present.

## Author contributions

**Conceptualization:** Chunlei Wang, Hengrui Chnag, Xianzhong Meng.

**Data curation:** Chunlei Wang, Hengrui Chnag.

**Formal analysis:** Chunlei Wang, Hengrui Chnag, Xianda Gao, Jiaxin Xu.

**Investigation:** Chunlei Wang, Jiaxin Xu, Xianzhong Meng.

**Methodology:** Chunlei Wang, Hengrui Chnag, Xianda Gao, Jiaxin Xu, Xianzhong Meng.

**Validation:** Xianda Gao.

**Visualization:** Chunlei Wang.

**Writing – original draft:** Chunlei Wang, Hengrui Chnag, Xianzhong Meng.

**Writing – review & editing:** Chunlei Wang, Xianda Gao, Xianzhong Meng.
